# Glasgow prognostic score for assessing the efficacy of whole-brain radiation therapy in cases of recursive partitioning analysis class 2 and class 3 multiple brain metastases: a retrospective study

**DOI:** 10.1007/s13760-023-02384-x

**Published:** 2023-09-25

**Authors:** Yojiro Ishikawa, Rei Umezawa, Takaya Yamamoto, Noriyoshi Takahashi, Kazuya Takeda, Yu Suzuki, Keita Kishida, So Omata, Satoshi Teramura, Kengo Ito, Takayuki Yamada, Keiichi Jingu

**Affiliations:** 1 Department of Radiation Oncology, Tohoku University Graduate School of Medicine, 1-1 Seiryo-chou, Aoba-ku, Sendai, Miyagi 980-8574 Japan; 2 Division of Radiology, Faculty of Medicine, Tohoku Medical and Pharmaceutical University, 1-15-1 Fukumuro, Miyagino-ku, Sendai, Miyagi 983-8536 Japan; 3 Department of Radiation Oncology, South Miyagi Medical Center, Ogawara, 989-1253 Japan

**Keywords:** Brain metastases, Glasgow Prognostic Score, Recursive partitioning analysis, Graded prognosis assessment, Radiosurgery

## Abstract

**Purpose:**

Whole-brain radiotherapy (WBRT) may not be beneficial for patients with brain metastases (BMs). The Glasgow Prognostic Score (GPS) is a suggested prognostic factor for malignancies. However, GPS has never been assessed in patients with BMs who have undergone WBRT. The purpose of this study was to determine whether GPS can be used to identify subgroups of patients with BMs who have a poor prognosis, such as recursive partitioning analysis (RPA) Class 2 and Class 3, and who will not receive clinical prognostic benefits from WBRT.

**Materials and methods:**

A total of 180 Japanese patients with BMs were treated with WBRT between May 2008 and October 2015. We examined GPS, age, Karnofsky Performance Status (KPS), RPA, graded prognostic assessment (GPA), number of lesions, tumor size, history of brain surgery, presence of clinical symptoms, and radiation doses.

**Results:**

The overall median survival time (MST) was 6.1 months. seventeen patients (9.4%) were alive more than 2 years after WBRT. In univariate analysis, KPS ≤ 70 (p = 0.0066), GPA class 0–2 (p = 0.0008), > 3 BMs (p = 0.012), > 4 BMs (p = 0.02), patients who received ≥ 3 Gy per fraction (p = 0.0068), GPS ≥ 1 (p = 0.0003), and GPS ≥ 2 (p = 0.0009) were found to significantly decrease the MST. Patients who had brain surgery before WBRT (p = 0.036) had a longer survival. On multivariate analysis, GPS ≥ 1 (p = 0.008) was found to significantly decrease MST.

**Conclusion:**

Our results suggest that GPS ≥ 1 indicates a poor prognosis in patients undergoing WBRT for intermediate and poor prognosis BMs.

## Introduction

The primary cause of death from cancer is metastasis [1]. Brain metastases (BMs) are the most common intracranial malignancies and occur in 10–40% of cancer patients [2, 3]. Whole-brain radiation therapy (WBRT) is effective for poor prognosis multiple BMs, defined as being recursive partitioning analysis (RPA) class 2 and class 3 [4, 5]; however, the use of WBRT has recently been decreasing because of improvements in stereotactic radiosurgery (SRS) and other techniques.

The QUARTZ (Quality of Life after Treatment for Brain Metastases) trial recently demonstrated that there was no difference between best supportive care (BSC) alone and BSC plus WBRT in non-small cell lung cancer (NSCLC) patients with poor prognostic factors in quality-adjusted survival years or overall survival (OS) [6]. WBRT for patients with a very poor prognosis may have little clinical utility, and BSC alone may be a more appropriate approach. Therefore, it is necessary to accurately identify patients who are unlikely to benefit from WBRT.

In previous studies, several patient factors were combined with BMs to define patients and groups with specific survival outcome. These prognostic classifications, which are mainly based on age, performance status, and condition of the primary tumor, are represented by RPA and diagnosis-specific graded prognosis assessment (DS-GPA) [4, 5]. However, it has been pointed out that conventional prognostic methods alone are inadequate for predicting short prognoses of BMs.

Recently, several studies have suggested that factors related to systemic inflammation, including the Glasgow Prognostic Score (GPS), are prognostic factors for various malignancies [7–10]. Although GPS was later modified (mGPS) due to isolated albumin decrements being uncommon, GPS is easy to measure, routinely available, and well standardized worldwide. It has been the subject of various prognostic studies for both operable and inoperable cancers [11]. Despite many patients with cancer being surveyed with results confirming the prognostic utility of GPS in different disease states, this has never been assessed in patients with BMs after WBRT.

The purpose of this study was to determine whether GPS can be used to identify subgroups of BMs that have a poor prognosis such as RPA Class 2 and Class 3, and will not receive clinical prognostic benefits from WBRT.

## Materials and methods

### Eligibility criteria

**Fig. 1:**
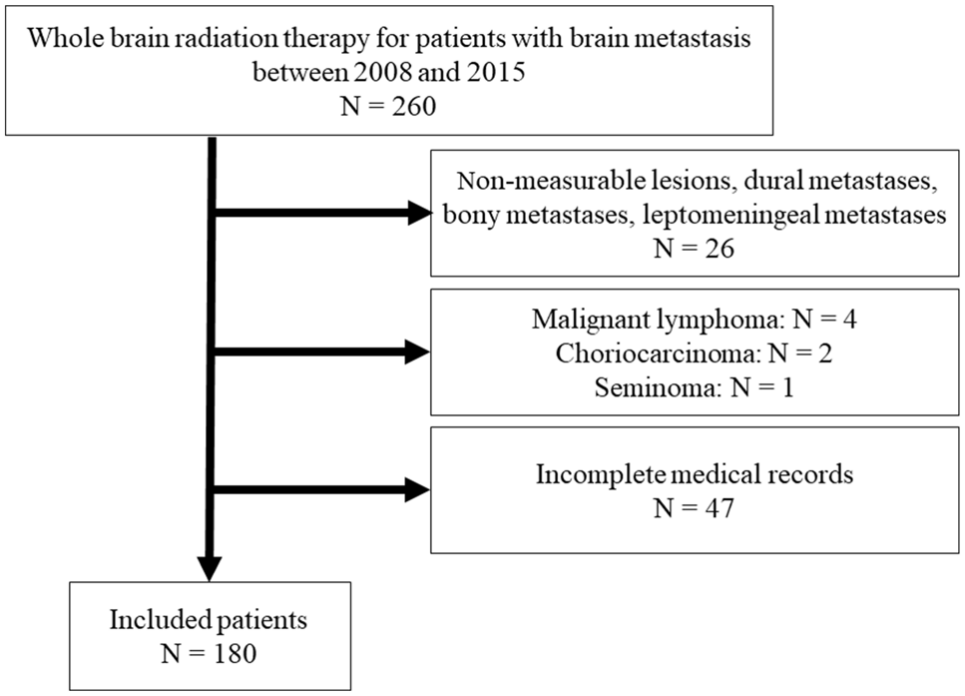
Flowchart of patients included in the study. There were 260 patients with brain metastases who were treated with whole-brain radiotherapy between 2008 and 2015 in our institution. Twenty-six patients were excluded because they had non-measurable lesions less than 5 mm, dural metastases, bony metastases, and leptomeningeal metastases. Four patients were excluded because they had malignant lymphoma. Additionally, two patients with choriocarcinoma and one patient with seminoma were excluded. Forty-six patients were excluded due to incomplete medical records (e.g., pretreatment blood tests not performed)

This retrospective study was carried out in compliance with the Declaration of Helsinki, and the institutional review board at XXXX approved this study. Between May 2008 and October 2015, we treated 180 Japanese patients with multiple BMs using three-dimensional conformal radiation therapy (3DCRT). We investigated the electronic medical records and radiotherapy records in our institution for patients with BMs. WBRT was performed patients with RPA class 2 or 3 [4]. In this study, patients with lesions that could not be measured were excluded. As a result, patients with only dural metastases or leptomeningeal metastases were excluded. Patients with multiple skull bone metastases were also excluded. Patients who had the diagnosis of the primary tumor confirmed by biopsy or imaging were eligible. Patients with diseases not specified in our institution’s treatment protocols or standard whole-brain irradiation protocols (e.g., ASTRO 2022) were excluded from the study [12]. Specifically, patients with malignant lymphoma, choriocarcinoma, and seminoma were excluded due to differences in irradiation coverage and dosing decisions determined by attending physicians. Figure 1 shows the exclusion criteria in this study.

Gadolinium-enhanced MRI was performed for each patient before WBRT, while MRI without contrast or CT with contrast was performed in resource-limited settings or for patients with contraindications to MRI. MRI with a 1.5 T MR imaging system included axial T1-weighted spin-echo, axial T2-weighted spin-echo and axial gadolinium-enhanced T1-weighted spin-echo sequences. Sequences such as fluid attenuated inversion recovery (FLAIR) and susceptibility weighted imaging (SWI) were included for some patients. One experienced neuroradiologist and one neuro-radiation oncologist were responsible for image analysis in this study.

### CT simulation and whole-brain radiation therapy procedure

All 3DCRT-treated patients were immobilized in the supine position. CT scans were performed at a slice thickness of 2.5 mm with a multidetector CT scanner (GE LightSpeed QX/i; GE Healthcare, Waukesha, WI, USA). The Eclipse™ treatment planning system (Varian Medical Systems, Palo Alto, CA, USA) was used for dose calculations during 3DCRT planning.

The clinical target volume (CTV) included the whole brain. The CTV was expanded in three dimensions with 0.5 cm margins to yield the planning target volume. In this study, a conventional WBRT field was used and hippocampal sparing techniques were not used. Patients were treated with 20–50 Gy using 10 MV photons with a conventional 2–4-field technique. Among the 180 patients included in this study, 106 (58.9%) were treated with 30 Gy/10 fractions, 43 (23.9%) were treated with 36 Gy/12 fractions, 19 (10.6%) were treated with 37.5 Gy/15 fractions, 10 (5.6%) were treated with 20–25 Gy/4–5 fractions, and two (1.1%) were treated with a dose between 40 and 50 Gy.

### Glasgow prognostic score evaluations

The GPS was obtained from the levels of CRP and Alb before WBRT and patients were divided into the following three score groups. Patients with both an elevated level of C-reactive protein (> 10 mg/l) and hypoalbuminaemia (< 35 g/l) were allocated to a GPS of 2. Patients in whom only one of those biochemical abnormalities was present were allocated to a group with GPS of 1. Patients in whom neither of those abnormalities was present were allocated to a group with GPS of 0 [7–11].

### Outcome assessments and follow-up

OS was defined as the time from the start of WBRT to the date of death from any cause. Follow-up assessments were conducted according to the attending physician's discretion and included blood sampling, CT scans of the trunk, and MRI or CT scans of the head every 1–3 months. These assessments were scheduled every 1–3 months during the first year and every 6 months thereafter.

### Statistical analysis

Statistical analyses were performed using JMP v16 (SAS Institute Inc., Cary, NC, USA). Statistical significance was set at p < 0.05 in all analyses. The optimal cutoff values for the number of BMs and the maximum diameter of metastases were obtained by calculating the area under the receiver operating characteristic curve (AUROC). The OS rates were determined using Kaplan–Meier estimates. The clinical parameters were investigated using univariate analysis (log-rank test). Hazard ratios with 95% confidence intervals (95% CIs) were calculated by using Cox’s proportional hazard regression model with the following variables: (i) KPS of > 70 at WBRT, (ii) RPA class 2, (iii) GPA ≥ 3, (iv) maximum tumor size ≥ 3.0 cm, (v) number of metastases ≥ 3 or ≥ 4, (vi) with brain surgery (vii) dose per fraction of WBRT, and (viii) GPS ≥ 1.

## Results

### Patient and brain tumor characteristics

Data for all of the 180 patients were included in the survival analysis. Table 1 shows the characteristics of the patients and the tumors. A total of 1,198 metastases were detected (median: 4.5 metastases per patient).

**Table 1:** Patient and tumor characteristics

	N or range	% or median
No. of patients	180	100%
Age (years)	11–83 (years)	Median 65.0 (years)
Male/Female	98/82	54.4%/45.6%
Karnofsky performance status		
30–40	16	8.9%
50–60	18	10.0%
70	50	27.8%
80	56	31.1%
90–100	36	20.0%
Missing	4	2.2%
Primary lesion site		
Lung	121	67.8%
Breast	26	14.4%
Gastrointestinal	12	6.7%
Other	20	11.1%
Histology		
Adenocarcinoma	96	53.3%
Squamous cell carcinoma	17	9.4%
Small cell carcinoma	21	11.7%
Invasive ductal carcinoma	20	11.1%
Sarcoma	4	2.2%
Other	2	1.1%
Missing	20	11.1%
RPA class		
II	91	50.6%
III	89	49.4%
GPA		
0–1	19	10.6%
1.5–2	98	54.4%
2.5–3	47	26.1%
3.5–4	16	8.9%
Number of lesions		
1	29	16.1%
2	20	11.1%
3	25	13.9%
4	16	8.9%
More than 5 lesions	90	50.0%
Tumor size (cm)	0.3–6.1 cm	median 1.72 cm
Primary lesion		
Controlled	11	6.1%
Uncontrolled	169	93.9%
Brain surgery		
With	17	9.4%
Without	163	90.6%
Stereotactic radiosurgery		
With	36	20.0%
Without	144	80.0%
Targeted therapy		
With	29	16.1%
Without	151	83.9%
Headache		
Absent	129	71.7%
Present	51	28.3%
Motor/Sensation deficit		
Absent	120	66.7%
Present	60	33.3%
Time interval from first diagnosis		
< 1 years	84	46.7%
≥ 1 years	96	53.3%
Edema peripheral brain tumors		
Absent	74	41.1%
Present	106	58.9%
Ascites/Pulmonary effusion		
Absent	136	75.6%
Present	44	24.4%
Use of steroid therapy		
Absent	104	57.8%
Present	76	42.2%
GPS		
0	74	41.1%
1	57	31.7%
2	49	27.2%

*RPA* Recursive partitioning analysis, *GPS* Glasgow prognostic score

### Survival outcomes

**Fig. 2:**
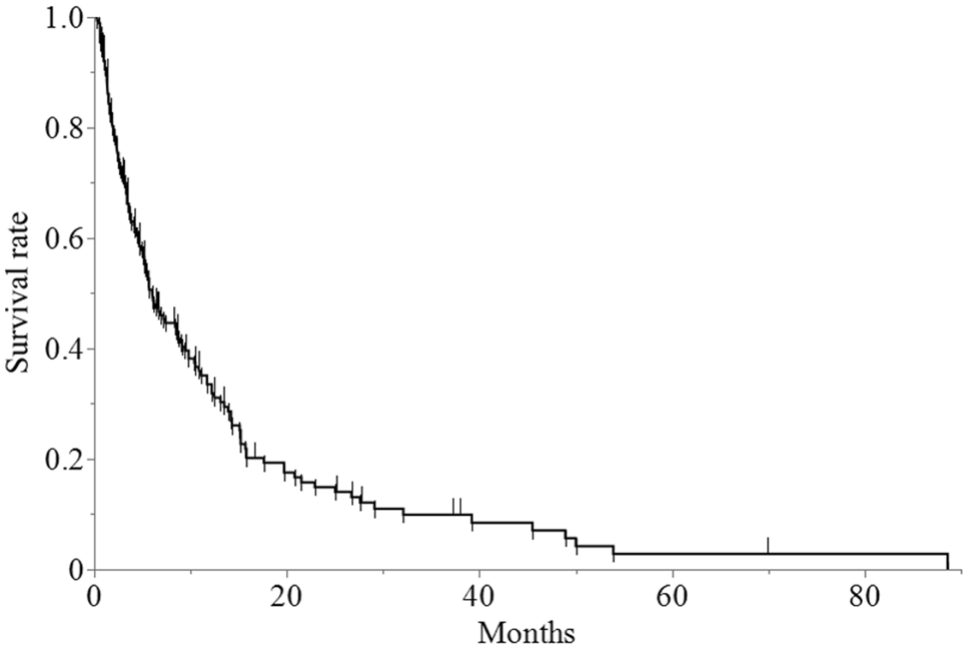
Overall survival rate after whole-brain radiation therapy for recursive partitioning analysis class 2 and class 3 multiple brain metastases

**Fig. 3:**
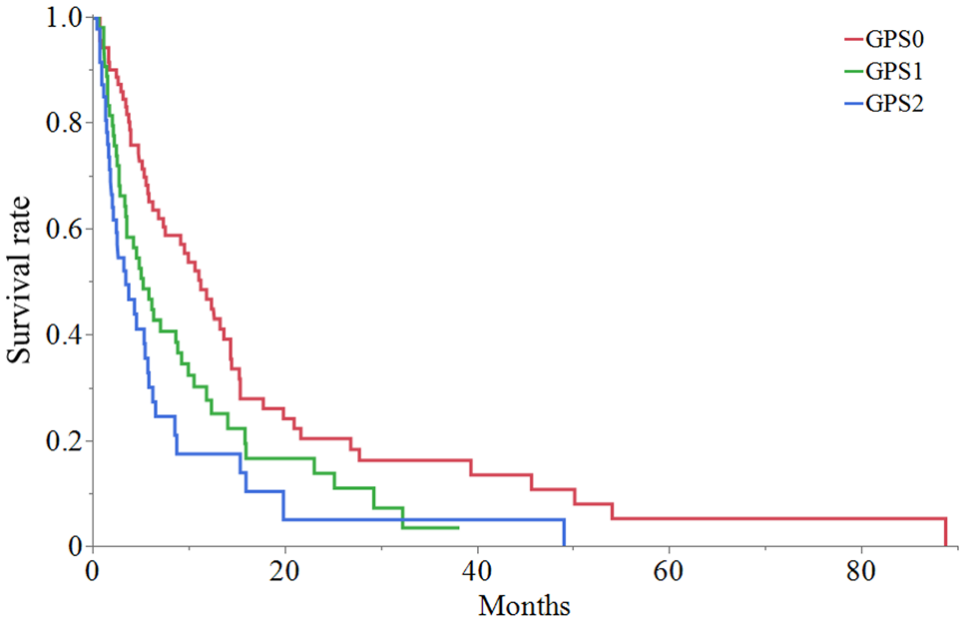
Comparative overall survival results, Glasgow Prognostic Score (GPS) group: 0 (red line), 1 (green line), and 2 (blue line) (color figure online)

The overall median survival time (MST) was 6.1 months (95% CI: 4.9–8.7 months) (Fig. 2). Patients with GPS 0, GPS 1 and GPS 2 had an MST of 11.1 months (95% CI: 6.7–14.2), 5.1 months (95% CI: 3.3–8.7) and 3.3 months (95% CI: 1.9–5.6), respectively (p = 0.004) (Fig. 3). Notably, 17 patients (9.4%) were alive more than 24 months after WBRT with a median survival period of 36 months (range: 24–37 months). These 17 patients included seven were men and ten were women. The median age of the patients was 59 years (range: 39–78 years). Nine patients were diagnosed with lung cancer, four were diagnosed with breast cancer, two were diagnosed with uterine cancer, one was diagnosed with rectal cancer, and one was diagnosed with cancer of an unknown primary origin. At the time of the initial diagnosis, five patients already had BMs. Among the lung cancers, eight were adenocarcinoma and one was small cell carcinoma. Molecularly targeted drugs were administered in four lung cancer patients. In 16 patients, there were five or fewer BMs. KPS was below 70 in six patients and it was above 70 in eleven patients. According to the RPA classification, there were eleven patients in class 2 and six patients in class 3. GPS was 1 or less in 16 patients, and only one patient had a GPS of 2. The median dose of WBRT was 36 Gy (range: 30–39 Gy). By the time of this prognostic study, eleven patients had passed away. Among the six patients who were still alive, four were in hospice care, one was disease-free, and one was undergoing chemotherapy for recurrent BMs. One patient showed an abscopal effect [13].

### Univariate and multivariate outcomes

In univariate analysis, Patients with RPA class 2 had an MST of 8.6 months (95% CI: 5.7–12.2) and patients with RPA class 3 had an MST of 4.7 months (95% CI: 2.7–6.1) (p = 0.019). KPS ≤ 70 at WBRT significantly decreased MST (4.1 [95% CI: 2.5–5.6] vs. 9.0 [95% CI: 6.1–12.2] months, p = 0.0066]). GPA 0–2 significantly decreased MST (5.0 [95% CI: 3.4–7.2] vs. 10.9 [95% CI: 5.7–13.9] months, p = 0.0008]). Having > 3 BMs, versus ≤ 3 BMs, decreased the survival period (5.3 [95% CI: 3.8–7.4] vs. 9.8 [95% CI 5.7–13.5] months, p = 0.012). Having > 4 BMs, versus ≤ 4 BMs, decreased the survival period (5.2 [95% CI: 3.6–7.4] vs. 8.4 [95% CI 5.7–11.7] months, p = 0.022).

A significant difference was found in MST between patients with and those without brain surgery before WBRT (12.2 [95% CI: 5.7–88.6] vs. 5.6 [95% CI: 4.4–8.5] months, p = 0.036). Although the total dose of WBRT (≥ 30 vs. < 30 Gy) was not a significant prognostic factor (p = 0.15), patients who received ≥ 3 Gy per fraction died sooner than did patients who received < 3 Gy per fraction (5.6 [95% CI: 4.2–6.9] vs.14.2 [95% CI: 5.6–27.6] months, p = 0.0068]. Furthermore, GPS ≥ 1 was significantly associated with decreased MST (4.2 [95% CI: 2.5–5.7] vs.9.8 [95% CI: 6.2–13.1] months, p = 0.0005).

Univariate analysis revealed that none of the following variables were significant prognostic factors: primary tumor site, histological type, presence of ascites or pleural effusion, steroid medications, surgery, and targeted therapy. Multivariate analysis showed that GPS ≥ 1 was significantly indicated poor prognosis. These results are shown in Table 2.

**Table 2:** Univariate and multivariate analysis of prognostic parameters after whole brain radiation therapy

	Univariate		Multivariate	
Parameter	Median OS Months (95% CI)	p-value	HR (95% CI)	p-value
Age				
60 years >	6.4 (4.2–9.4)	0.45		
60 years ≤	5.7 (4.6–10.9)			
Karnofsky performance status				
70 >	9.0 (6.1–12.2)	0.0066	0.95 (0.51–1.76)	0.87
70 ≤	4.1 (2.5–5.6)			
RPA				
Class II	8.6 (5.7–12.2)	0.019	0.84 (0.45–1.57)	0.18
Class III	4.7 (2.7–6.1)			
GPA				
0–2	5.0 (3.4–7.2)	0.0008	0.73 (0.46–1.16)	0.19
3–4	10.9 (5.7–13.9)			
GPS				
≥ 1	4.4 (3.1–5.7)	0.0003	0.62 (0.41–0.95)	0.0288
< 1	11.1 (7.2–14.2)			
GPS				
≥ 2	3.3 (1.8–5.3)	0.0009	0.68 (0.43–1.06)	0.09
< 2	8.5 (5.7–11.1)			
Number of lesions				
3 ≥ 3 < 4 ≥ 4 < 5 ≥	5.3 (3.8–7.4) 9.8 (5.7–13.5) 5.2 (3.6–7.4) 9.1 (5.7–11.7) 5.6 (3.6–8.7)	0.012 0.020 0.15		
5 <	6.2 (4.7–9.8)		1.09 (0.57–2.07)	0.8
10 ≥	5.6 (2.3–12.5)	0.62		
10 <	6.2 (4.9–9.4)		1.34 (0.72–2.51)	0.35
Tumor size				
2.0 cm ≥	5.6 (3.6–8.5)			
2.0 cm <	7.2 (5.0–10.5)	0.71		
1.0 cm ≥	5.6 (3.6–6.4)			
1.0 cm <	9.8 (5.2–13.9)	0.27		
Brain surgery				
With	12.2 (5.7–88.6)	0.036	0.63 (0.32–1.24)	0.18
Without	5.6 (4.7–8.5)			
Stereotactic radiosurgery				
With	6.1 (4.1–13.5)	0.94		
Without	6.1 (4.4–9.1)			
Targeted therapy				
With	6.7(4.4–12.5)	0.1		
Without	4.2 (3.3–5.6)			
Use of steroid therapy				
Yes	5.6 (3.3–8.7)	0.55		
No	6.9 (4.7–10.5)			
Headache Present	6.7 (4.4–10.4)	0.99		
Absent	5.6 (4.4–8.5)			
Motor/sensation deficit				
Present	4.1 (3.1–6.1)	0.25		
Absent	7.4 (5.4–10.5)			
Hippocampal metastases				
With	6.4 (0.3–14.2)	0.18		
Without	6.1 (5.0–8.7)			
Limbic system metastases				
With	3.8 (2.4–11.7)	0.50		
Without	6.1 (5.1–9.1)			
Time interval				
1 years	6.0 (4.1–7.2)	0.31		
≥ 1 years	8.4 (4.6–11.7)			
Edema peripheral brain tumors				
Present	5.7 (4.1–8.7)	0.67		
Absent	6.9 (4.6–12.5)			
Ascites/Pulmonary effusion				
Present	5.2 (3.1–11.7)	0.74		
Absent	6.1 (4.9–9.0)			
Total dose				
≥ 30 Gy	9.4 (5.6–12.2)	0.15		
< 30 Gy	5.2 (3.8–6.2)			
Dose per fraction				
≥ 3 Gy < 3 Gy	5.6 (4.2–6.9) 14.2 (5.6–27.6)	0.0068	0.62 (0.37–1.05)	0.078

## Discussion

Glasgow Prognostic Score (GPS) is an index that combines serum CRP and serum Alb levels, and it is a known prognostic factor for nonsmall cell lung cancer [11]. GPS has also been shown to provide additional prognostic information for patients with advanced cancer in various organs [7–10]. Previous studies have revealed that low serum Alb levels signify poor prognosis in patients with BMs [14, 15]. Therefore, it is reasonable to consider GPS as an important clinical prognostic factor in patients with BMs.

GPS has not been reported as a prognostic factor after WBRT for BMs. Patients with GPS ≥ 1 had a significantly shorter survival period than did patients with GPS < 1 in this study. WBRT is a form of palliative therapy, and patients undergoing WBRT are at high risk of high CRP and low Alb levels. Patients with BMs are also at risk for systemic inflammation due to prolonged use of chemotherapy and systemic metastases. Hence, it may be beneficial to evaluate GPS as a prognostic factor before WBRT. It is not unexpected that GPS serves as an indicator of overall physical status and exerts an influence on OS. However, from a clinical perspective, for example, it could be beneficial in patients when assessment of RPA or DS-GPA becomes challenging. It is notable that the majority of the patients with long-term survival had a GPS of 1 or less.

According to the QUARTZ trial, WBRT for patients with brain metastases from NSCLC provided no additional clinically significant benefit [6]. The present study included patients with a relatively poor prognoses, and the GPS ≥ 1 patient group might have included patients for whom WBRT can be avoided.

Some extracranial tumor factors are associated with the prognosis for patients with BMs. One significant prognostic factor is primary tumor control [4, 24]. In our study, the primary tumor was controlled in 11 (5.6%) of the 180 patients, a percentage that is similar to that in another study [17]. Gaspar et al. reported that the median survival periods after WBRT were 7.1, 4.2, and 2.3 months for patients with RPA class 1, class 2, and class 3, respectively [4]. Although recent advances in treatment techniques have improved outcomes for patients in each class, the prognosis generally remains poor [18]. In our study, the OS periods for RPA class 2 and class 3 patients were 8.6 months (95% CI: 5.7–12.2) and 4.7 months (95% CI: 2.7–6.1), respectively (p = 0.019). OS periods tended to be similar to or slightly longer than those previously reported. In this study, histology and the presence of ascites or pleural effusion were not found to be significant prognostic factors; however, the reasons for this were not clear. Given that this study focused on the poor prognosis group, it is plausible that a large number of patients were in the advanced stages of their disease, for which the impact of histological type might be less prominent. While pleural fluid and ascites did not emerge as prognostic factors, it is notable that GPS might be influenced by the inclusion of albumin levels, which are connected to colloid osmotic pressure [19]. However, we acknowledge that delving into this topic within this study might be challenging.

KPS, RPA, and GPA were shown to be significant prognostic factors in our univariate analysis, as was found in studies by the Radiation Therapy Oncology Group (RTOG) [4, 24]. The fact that these factors did not show significant differences in our multivariate analysis may be due to the inclusion of many poor prognosis BMs in the present study [20].

DS-GPA has been proposed for lung cancer, breast cancer, gastrointestinal cancer, renal cancer, and malignant melanoma [5]. In recent years, the prognosis of lung cancer has depended on the presence or absence of EGFR mutation and ALK fusion protein [21]. In our study, 121 patients (67.8%) had lung cancer, and those patients included 82 patients (42.5%) with lung adenocarcinoma. Targeted therapy was performed in 29 patients (16.1%) but it was not a clear prognostic factor in our univariate analysis.

Various radiation doses including 20 Gy/4–5 fractions, 30 Gy/15 fractions, 30 Gy/10 fractions, 37.5 Gy/15 fractions, and 40 Gy/20 fractions are used for WBRT [22]. Although we found no significant difference in survival between the various total doses in our study, a dose of 40 Gy/20 fractions was found to be significantly superior over 20 Gy/4–5 fractions for intracranial tumor control in two randomized controlled trials [23–25]. Good prognosis has been reported in patients who received WBRT at a dose of 2–2.5 Gy for breast cancer [26]. The present study included 26 breast cancer patients (14.4%), and that may be the reason for the significant difference in univariate analysis. Concerning dose fractionation for WBRT, it is important to discuss how to select the dose fraction, and selection bias might therefore be one of the causes of the difference in prognosis in this study.

This study had some limitations. Since this study was retrospective and conducted only for patients in whom WBRT was performed, the possibility of bias in the selection of patients for analysis of the OS rate cannot be ruled out. Moreover, since only OS was evaluated in our study, it might represent the prognosis of patients eligible for WBRT rather than the efficacy of WBRT. In multivariate analysis, only GPS (≥ 1 vs. < 1) showed a significant difference in MST; however, we could not find a clear reason for there being a significant difference with GPS only. This study included relatively young patients. However, it was not possible to analyze the prognostic impact of these younger patients. All the patients predate the use of standardized criteria such as response assessment in neuro-oncology brain metastases criteria, and tumor response was difficult to assess and progression-free survival was not assessed, which was an important drawback. The total doses varied and some patients included doses as high as 40–50 Gy, which are not used these days. Additionally, a common complication following WBRT is neurocognitive function (NCF) impairment, which can decrease the quality of life for patients after WBRT [24]. Many of the patients in this study were elderly, and NCF was not assessed before treatment. Therefore, cognitive function after WBRT, which is a clinically important indicator, could not be analyzed.

## Conclusion

The aim of this study was to determine whether GPS can be used to identify subgroups of patients with BMs who have intermediate and poor prognoses and would not benefit from WBRT to improve prognosis. Our findings suggest that a GPS score of ≥ 1 is associated with a poor prognosis in patients undergoing WBRT. WBRT does not improve prognosis beyond its palliative effects in patients with a GPS score of 1 or higher, and treatment with BSC alone may be considered an option for patients with poor prognosis. Prospective research is needed to confirm the results of this study. Furthermore, investigating the relationship between GPS and the prognosis of WBRT for different diseases, such as lung and breast cancers, will be necessary in the future.

## Data Availability

The datasets analyzed during the current study are available from the corresponding author on reasonable request.

## Abbreviations

Alb: Albumin
AUROC: Area under the receiver operating characteristic curve
BMs: Brain metastases
CTV: Clinical target volume
CT: Computed tomography
DS-GPA: Diagnosis-specific graded prognosis assessment
FLAIR: Fluid Attenuated Inversion Recovery
GPS: Glasgow Prognostic Score
HMs: Hippocampal metastases
KPS: Karnofsky performance status
MRI: Magnetic resonance imaging
mGPS: Modified Glasgow Prognostic Score
NCF: Neurocognitive function
OS: Overall survival
RPA: Recursive partitioning analysis
RTOG: Radiation Therapy Oncology Group
SRS: Stereotactic radiosurgery
SWI: Susceptibility weighted imaging
WBRT: Whole-brain radiotherapy
3DCRT: Three-dimensional conformal radiation therapy
